# Deep mowing rather than fire restrains grassland *Miscanthus* growth *via* affecting soil nutrient loss and microbial community redistribution

**DOI:** 10.3389/fpls.2022.1105718

**Published:** 2023-01-13

**Authors:** Zhuxin Mao, Yuchao Wang, Qian Li, Weimin Li, Hong Wang, Yang Li, Ming Yue

**Affiliations:** ^1^ Xi’an Botanical Garden of Shaanxi Province/Institute of Botany of Shaanxi Province, Xi’an, China; ^2^ Key Laboratory of Resource Biology and Biotechnology in Western China, Northwest University, Xi’an, China

**Keywords:** fire, deep mowing, *Miscanthus*, soil nutrient limitation, microbial community

## Abstract

Fire and mowing are crucial drivers of grass growth. However, their effects on soil properties, microbial communities, and plant productivity in dry-alkaline grasslands have not been well investigated. This study evaluated the effects of mowing (slightly and deeply) and fire on vegetation traits (Tiller number per cluster and plant height) and biomass (plant dry weight), and soil availability of N, P, and K, as well as soil microorganism abundance in a *Miscanthus* system. We designed one control and three experimental grass plots (slightly and deeply mowed, and burned) in 2020–2021 in the Xi’an Botanical Garden of Shaanxi Province, Xi’an, China. Tiller number, plant height per cluster, and soil N, P, and K availability during *Miscanthus* growth decreased significantly (*p* < 0.05) in all treatments compared to the control. However, this effect was much greater in the deep-mowing plot than in the other plots. After harvest, deep mowing induced the greatest effect on biomass among all treatments, as it induced a 5.2-fold decrease in dry biomass relative to the control. In addition, both fire and mowing slightly redistributed the community and diversity of the soil bacteria and fungi. This redistribution was significantly greater in the deep-mowing plot than in other plots. In particular, relative to the control, deep mowing increased the abundance of *Firmicutes* and especially *Proteobacteria* among soil bacterial communities, but significantly (*p* < 0.05) decreased *Basidiomycota* and increased *Ascomycota* abundance among soil fungal communities. We conclude that nutrient limitation (N, P, and K) is crucial for *Miscanthus* growth in both mowing and fire grasslands, whereas deep mowing can induce soil nutrient loss and microorganism redistribution, further restraining grass sustainability in dry-alkaline grasslands.

## 1 Introduction

Grassland covers a large area of the planet, accounting for 24% of the global vegetative land area (Ren et al., 2008). In China, the total area is approximately 400 million hectare, contributing more than 40% of the total land area (Zhao and Li, 2018). Despite their importance, grasslands face a high threat, as they have been rapidly declining in the past decades due to biodiversity loss (Carbutt et al., 2017; Wang et al., 2020a). Both the dynamics and diversity of grassland ecosystems are easily affected by natural and artificial disturbances such as fire, grazing, and mowing (Loram-Lourenco et al., 2020; Vermeire et al., 2020; Slette et al., 2021; Qin et al., 2022). These disturbances alter the plant community structure and ecosystem function of grasslands as they are managed at inappropriate scales, intensities, and frequency levels, thereby leading to their degradation (Lu et al., 2012; Zhang et al., 2020). In the past decade, prescribed burning and mowing have become important tools for managing and restoring grassland ecosystems in China (Lu et al., 2012; Zhu et al., 2021), even though they significantly impact grasslands’ soil properties, plant growth, and nutrient cycles (Dowhower et al., 2021), further disturbing various ecological processes, such as herbivory and litter decomposition (Lu et al., 2012; Bai and Cotrufo, 2022). Few studies have investigated the effects of fire and mowing on the microbial community structure in grassland ecosystems, despite the fact that microorganisms play key roles in regulating the nutrient cycle and carbon fixation (Zhao et al., 2020; Bai and Cotrufo, 2022).

Prescribed burning is a major strategy used worldwide to manage grassland ecosystems, as it directly drives the use of natural resources, such as water, light, and nutrients, by native plants (Fidelis et al., 2012; Gordijn and O'Connor, 2021). Plant physiological processes that require water, energy, and nutrients are altered by prescribed burning (Lu et al., 2012; Boughton et al., 2013), and plant nutrition is a key feature affected by the fire in grasslands (Fultz et al., 2016). Reinhart et al. (2016) reported that nutrient concentrations (N and P) were much higher in burned soils than in the control soils. Variations in the nutrient cycle in response to prescribed burning may result in changes in the primary productivity of grasslands (Fidelis et al., 2012). Furthermore, previous studies have focused on the effect of fire on plant diversity and soil nutrients, whereas its effects on microbial community structure in grasslands are still not well investigated (Lu et al., 2012; Wragg et al., 2018; Vermeire et al., 2020). Soil microorganisms have greater potential to affect nutrient mobility. They may also compete with plants for the same limiting nutrients in grasslands (Bing et al., 2016; Yang et al., 2020a; Bonanomi et al., 2022). Predicting the complex response of biodiversity to fire disturbance are also dependent on the microbial and functional groups in soils (Farnsworth et al., 2014). Thus, a better understanding of the relationships among plant growth, soil nutrients, and microbial communities under the prescribed burning treatment is needed to provide a context for the sustainable development of grassland ecosystems (Gordijn and O'Connor, 2021).

Mowing, the mechanical removal of vegetation, is considered an effective management practice in grasslands, which has characteristics similar to those of prescribed burning (Vermeire et al., 2020; Török et al., 2021). Compared with fire, mowing requires less planning and presents low ecological risk (Fidelis et al., 2012). Another benefit is that mowing increases plant diversity in grassland ecosystems by promoting aboveground conditions, besides preserving the high conservation value of grassland ecosystems (Fidelis et al., 2012; Vermeire et al., 2020). Therefore, mowing may be an attractive alternative to prescribed burning for vegetation management. However, plant productivity and soil nutrients are key factors that potentially limit the response of ecosystems to mowing (Chen et al., 2021). Yang et al. (2020b) found that mowing had no significant influence on soil nutrient concentrations; however, plant N and P content were increased by mowing. Soil microorganisms can participate in the biogeochemical cycles of various nutrients (N and P) in grassland ecosystems (Bing et al., 2016; Wang et al., 2020b; Wilcox et al., 2022). However, the potential influence of mowing on the microbial community in grasslands is not well understood.

*Miscanthus*, a perennial herb widely distributed in western China, is the main forage in summer grasslands and forest steppes. It plays a crucial role in sustaining the ecosystem functions of grasslands. The goal of this study was to evaluate the effects of prescribed burning and mowing on plant growth, soil properties, and microbial communities in grassland ecosystems. The specific aims were to 1) assess their effects on plant growth and soil properties, 2) further indicate their effects on microbial communities (bacteria and fungi), and 3) analyze the internal relations of plant biomass, soil properties, and microbial community.

## 2 Materials and methods

### 2.1 Experimental site

The experimental site is located in Xi’an Botanical Garden of Shaanxi Province (34°21′ N, 109°03′ E), Xi’an, China. This study site is characterized by rainy summer and snowy winter. The annual mean temperature was 14.1°C, and the monthly mean temperature ranged from 4.1°C in January to 30.2°C in July. The annual mean precipitation is 709 mm, with 59% of the rainfall occurring from July to October. The area covered by *Miscanthus* grass was 1650 m^2^.

### 2.2 Experimental design and sampling

**Figure 1:**
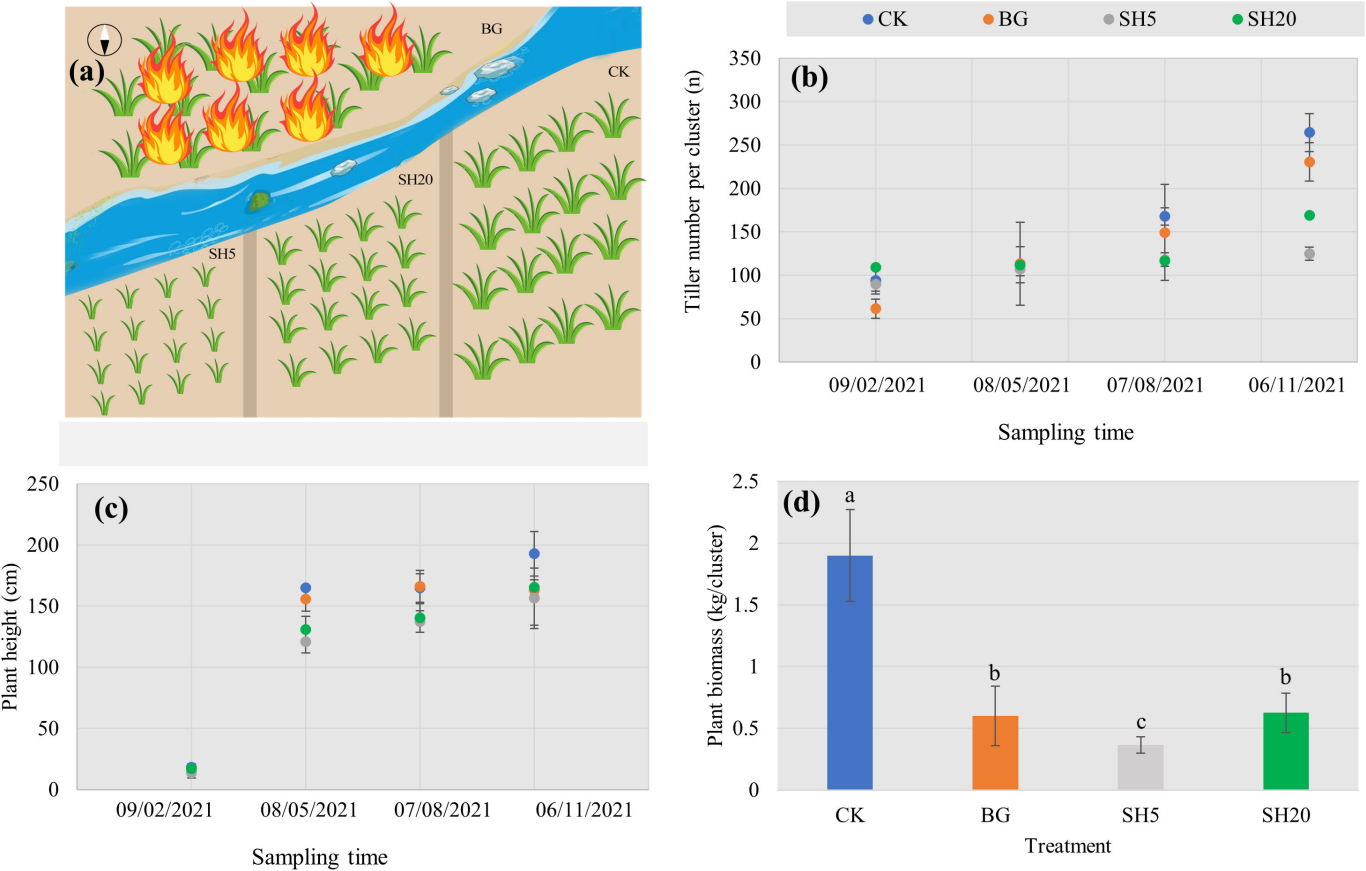
Concept map of experimental design with four treatments of control (CK), slightly (SH5) and deeply mowed (SH20), and burned grass plot (BG) **(A)**, and their effects on traits of *Miscanthus* with the growth period: Tiller number per cluster **(B)**, Plant height with different treatments **(C)**, and *Miscanthus* biomass (dry weight per cluster) after harvest **(D)**. Within each subgraph according to Tukey’s mean separation test, values with the same lower case presents a non-significant difference (*p* = 0.05), while different lower-case letters show a significant (*p* < 0.05) difference.

At the end of 2020, the selected *Miscanthus* growing area at four years-old was divided into four plots of 10 × 10 m^2^, each threat of which had three replicates. As being illustrated in (**Figure 1A**), these plots were treated as follows: without any practices as a control (CK), with 5-cm stubble height by mowing (SH5), with 20-cm stubble height by mowing (SH20), and burned *in situ* (BG). All the treatments were performed under natural conditions.

In 2021, all four treatments during *Miscanthus* growth were measured both the number of buds and plant heights in February, May, August, and November, respectively. At the end of November, we harvested all four treatments to determine their respective aboveground biomass, while there were left the stubble with a 2cm-height. The plant samples were collected by hand mowing during a vigorous growth period. Plant samples were collected by hand during the vigorous growth period, and then taken to the laboratory for oven drying (65°C for 24 h) to determine the dry plant biomass. After removing the upper litter in each of the four treatments, we collected five soil subsamples using a drill to mix one sample, and four samples were taken randomly from each treatment (total of 16 samples in the four treatments). All soil samples were sieved through a 2-mm mesh, transported back to the laboratory, and then divided into two subsamples: one was stored at –80°C for the analysis of soil microbial diversity, and the other was stored at 4°C to measure soil properties. After removing stones and plant and animal debris, the soil samples were dried and weighed to obtain the dry weight of each pot. The air-dried soil samples were ground, passed through a 100-mesh (0.15 mm) nylon screen, and stored in the dark at a low temperature for soil physicochemical analysis.

### 2.3 Plant traits

In 2021, the following plant traits of all four treatments during *Miscanthus* growth in February, May, August, and November were measured: tiller number per cluster and plant height per cluster. Each trait was replicated five times for each treatment.

### 2.4 Determinations of soil physical-chemical properties

Soil pH was measured with a pH meter in a 1:2.5 (m:v) soil-to-water extract using the potentiometric method. The total soil organic carbon (SOC) was determined using the potassium dichromate oxidation heating method. Total nitrogen (TN) was determined by microwave digestion and the *Kjeldahl* method. Total phosphorus (TP) and total potassium (TK) were determined by the sodium hydroxide melt-molybdenum antimony colorimetric method and the sodium hydroxide melt-flame photometry method, respectively. The measurement of soil available nutrients was performed following Lu (2000), i.e., alkali-hydrolyzed nitrogen (AN), available phosphorus (AP), and available potassium (AK) were measured using the alkali solution diffusion method, the spectrophotometric colorimetry method (Olsen, 1954), and flame photometry, respectively.

### 2.5 Extraction, amplification, and sequencing of soil sample DNA

Following the manufacturer’s instructions, microbial DNA was extracted from soil samples (0.5 g) in triplicate using the E.Z.N.A.^®^ soil DNA Kit (Omega Bio-tek, Norcross, GA, U.S.). An ABI GeneAmp^®^ 9700 polymerase chain reaction (PCR) thermocycler (ABI, CA, USA) (Yusoff et al., 2013) was used to amplify the V4-V5 region of bacterial 16S rRNA genes with primer pairs 515F (5’-GTGCCAGCMGCCGCGG-3’) and 907R (5’-CCGTCAATTCMTTTRAGTTT-3’). The PCRs were performed in a 20-μL reaction with the mixtures of 5 × *TransStart* FastPfu buffer (4 μL), 2.5 mM dNTPs (2 μL), 5 μM primer (0.8 μL), *TransStart FastPfu* DNA polymerase (0.4 μL), template DNA (10 ng), and double-distilled water (10 μL). The PCR thermal cycling program was as follows: initial denaturation (95°C, 3 min), followed by 27 cycles at 95°C (30 s), 55°C (30 s), and 72°C (45 s), a final extension at 72°C (10 min), and ending at 4°C. The PCR products were isolated using agarose gel (2%), purified by AxyPrep DNA Gel Extraction Kit (Axygen Biosciences, Union City, CA, USA), and then quantified by Quantus™ Fluorometer (Promega, USA). Finally, the NCBI short-read archive with Bioproject ID (PRJNA765434) was used to deposit raw amplicon sequences.

Following the method described by Adams et al. (2013), ITS1F (5′-CTTGGTCATTTAGAGGAAGTAA-3′) and ITS2 (5′-GCTGCGTTCATCGATGC-3′) primers were used to amplify the fungal ITS1 region. PCR was performed as described for the bacterial 16S rRNA gene amplification. Equal amounts of purified amplicons with paired-end sequencing (2 × 300 bp) were carried out using standard protocols (Majorbio Bio-Pharm Technology Co. Ltd., Shanghai) on the Illumina MiSeq platform (Illumina). Raw reads were deposited into the NCBI Sequence Read Archive (SRA) database.

### 2.6 Bioinformatic analyses

Following the method of Magoč and Salzberg (2011), the Illumina sequencing data were analyzed using Flash with the option max-overlap 200, while the unassembled sequences were removed. FASTQ files were generated and implemented in QIIME 1.9.1, as described by Caporaso et al. (2010). Bacterial sequences were searched against the Ribosomal Database Project Classifier to identify and discard chimeric sequences (Schloss et al., 2009). Operational taxonomic units (OTUs) with 97% similarity cutoff were clustered using UPARSE (version 7.1), and the taxonomy of each OTU representative sequence was analyzed against the 16S rRNA database (Silva) using a confidence threshold of 0.7. Fungal ITS sequences were assigned to taxa by using a naive Bayesian classifier (Abarenkov et al., 2010). OTUs with 97% similarity cutoff were clustered following previous studies (Caporaso et al., 2010; Edgar, 2013). OTUs of the bacterial and fungal datasets with low abundance were filtered and discarded following the OTU table (Brown et al., 2015; Oliver et al., 2015). Alpha diversity of bacterial and fungal communities (i.e., OTU richness, Sobs, Shannon’s diversity index, Shannon and Shannon’s evenness index, Shannon-even) were calculated using the method described by Schloss et al. (2009).

### 2.7 Data analysis

A one-way analysis of variance (ANOVA) followed by Tukey’s *post hoc* test (p < 0.05) was used to test the effects of treatments on plant traits, soil properties, and soil microbial community composition. Before statistical analyses, all data were tested for both variance homogeneity and distribution normality, and log-transformation analysis was used when necessary.

Non-metric multidimensional scaling (NMDS) was used to visualize differences in microbial community composition among treatments using the Bray-Curtis dissimilarity matrix. Based on the Bray-Curtis distance matrixes with 9999 permutations (with the Adonis function), permutational multivariate analysis of variance (PERMANOVA) was carried out to test the respective effects of treatments on the soil microbial communities. Redundancy analysis (RDA) and the heatmap function were used to visualize the correlations between plant traits, soil properties, and microbial community composition. In addition, relationships between microbial alpha diversity, soil properties, and plant traits were determined using Pearson correlation analysis. All analyses were performed using R version 3.6.2.

## 3 Results

### 3.1 Variations of *Miscanthus* traits with four treatments

The variation in the selected *Miscanthus* traits with four treatments over time is shown in **Figure 1**. In all treatments, both tiller number per cluster and plant height showed a natural increasing trend with *Miscanthus* growth (**Figures 1B, C**). However, the tiller number per cluster of *Miscanthus* in the mowed plot and burned plot was altered and significantly (*p* < 0.05) less than that of the control at the end of growth. Plant height in both the mowed and burned plots was lower than that of the control at the end of *Miscanthus* growth, but not significantly. After harvest, the dry plant weight per cluster in both the mowed and burned plots was significantly lower than that of the control. In particular, the dry weight showed a 5.2-fold decrease in the SH5 plot relative to the control (**Figure 1D**).

### 3.2 Variation of soil physical-chemical properties with four treatments

The selected properties of soils with four treatments (CK, BG, SH5, and SH20) for *Miscanthus* growth (soil pH and total concentration of SOC, N, P, and K) are shown in **Table 1**. Relative to the control plot, the soil pH (0–20 or 20–40 cm) in both the mowed and fire plot was not significantly (*p* < 0.05) altered and ranged from 7.73 to 8.00. Similarly, both mowing and fire did not significantly alter the total concentration of SOC, N, P, and K in neither the 0–20 nor the 20–40 cm layer.

**Table 1:** Selected properties (pH, total concentration of soil organic carbon [SOC], N, P, and K) of soil under four treatments.

Sampling date	Treatment	Soil layer	pH		SOC		TN		TP		TK	
		cm	mean	s.d	mean	s.d	mean	s.d	mean	s.d	mean	s.d
**09/02/2021**	CK	0–20	7.78	0.11	18.20	0.80	1.69	0.06	0.68	0.00	20.53	0.18
		20–40	7.82	0.15	17.59	1.40	1.44	0.08	0.74	0.00	20.31	0.28
	BG	0–20	7.76	0.11	16.28	0.20	1.83	0.13	0.65	0.00	20.68	0.23
		20–40	7.86	0.16	14.95	1.15	1.50	0.13	0.76	0.01	20.46	0.37
	SH5	0–20	7.82	0.09	15.63	1.01	1.59	0.12	0.71	0.00	20.51	0.32
		20–40	7.93	0.13	14.70	1.00	1.37	0.14	0.67	0.01	20.40	0.18
	SH20	0–20	7.76	0.16	19.97	0.10	1.66	0.04	0.67	0.01	20.41	0.56
		20–40	7.67	0.07	21.53	0.06	1.44	0.11	0.78	0.00	20.06	0.57
**08/05/2021**	CK	0–20	7.86	0.14	18.20	0.80	1.47	0.04	0.67	0.00	18.67	0.21
		20–40	7.89	0.10	17.59	1.40	1.22	0.11	0.70	0.00	18.55	0.19
	BG	0–20	7.81	0.15	15.89	0.25	1.55	0.05	0.73	0.01	18.87	0.10
		20–40	7.94	0.15	14.31	0.47	1.29	0.04	0.81	0.01	18.71	0.17
	SH5	0–20	7.87	0.14	17.61	1.17	1.64	0.06	0.68	0.01	18.54	0.15
		20–40	7.97	0.10	15.06	0.15	1.50	0.07	0.66	0.01	18.24	0.06
	SH20	0–20	7.72	0.13	17.96	1.32	1.81	0.10	0.68	0.00	18.40	0.03
		20–40	7.90	0.20	19.79	0.69	1.69	0.02	0.71	0.01	18.47	0.15
**07/05/2021**	CK	0–20	7.70	0.12	19.09	0.09	1.62	0.02	0.68	0.01	19.51	0.21
		20–40	7.89	0.12	17.63	0.21	1.18	0.05	0.69	0.01	19.34	0.24
	BG	0–20	7.86	0.11	15.05	1.06	1.35	0.03	0.69	0.00	19.53	0.20
		20–40	7.86	0.16	13.81	0.94	1.16	0.03	0.72	0.01	19.59	0.12
	SH5	0–20	7.81	0.16	15.32	1.25	1.37	0.02	0.68	0.00	19.24	0.10
		20–40	8.00	0.17	14.21	1.36	1.17	0.01	0.67	0.02	19.59	0.41
	SH20	0–20	7.77	0.06	16.81	1.04	1.51	0.04	0.69	0.01	19.55	0.14
		20–40	7.88	0.14	19.18	0.14	1.61	0.04	0.73	0.01	19.62	0.16
**06/11/2021**	CK	0–20	7.81	0.01	18.38	0.94	1.55	0.09	0.67	0.01	19.37	0.29
		20–40	7.85	0.03	18.54	0.11	1.28	0.04	0.73	0.01	18.74	0.30
	BG	0–20	7.81	0.01	15.90	1.01	1.60	0.04	0.68	0.01	19.01	0.30
		20–40	7.91	0.01	15.09	0.30	1.37	0.03	0.66	0.01	18.79	0.12
	SH5	0–20	7.75	0.02	17.79	1.27	1.79	0.01	0.68	0.01	18.92	0.10
		20–40	7.94	0.02	16.91	0.11	1.28	0.04	0.76	0.00	18.52	0.19
	SH20	0–20	7.73	0.00	17.68	0.92	1.51	0.03	0.68	0.01	18.86	0.26
		20–40	7.93	0.01	18.08	0.82	0.95	0.03	0.66	0.01	18.65	0.26

**Figure 2:**
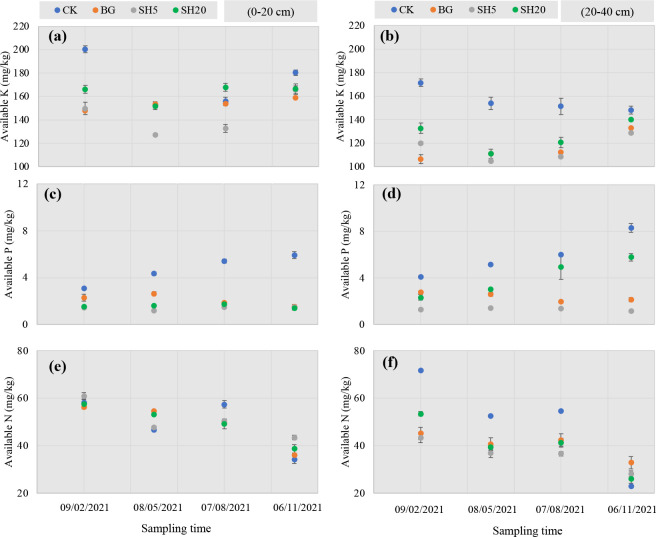
Variation of available K **(A, B)**, P **(C, D)**, and N **(E, F)** in soils under different treatments (left, 0–20 cm soil layer; right, 20–40 cm soil layer) over *Miscanthus* growth from 09/02/2021 to 06/11/2021.

Variations in available K, P, and alkali-hydrolyzed N in soils with four treatments over *Miscanthus* growth are shown in **Figure 2**. For available soil K, both mowing and fire significantly reduced its concentration in both the 0–20 cm and 20–40 cm layers, while this concentration was much greater in the 0–20 cm layer than in the–20–40 cm layer. In the 0–20 cm layer during *Miscanthus* growth, the concentration of available K in the soil quickly decreased from 09/02/2021 to 08/05/2021 and then slightly increased until 06/11/2021. This trend was similar to that of the 20–40 cm soil layer in all treatment plots, except for the control plot, in which soil available K slightly decreased from 09/02/2021 to 06/11/2021 with *Miscanthus* growth. For available soil P, both mowing and fire significantly (p < 0.05) decreased its concentration in both soil layers relative to the control. In both soil layers, soil P concentration in all treatment plots increased with *Miscanthus* growth, and this trend was much larger in the control plot than in the other plots. Unlike soil available P, soil available N slowly decreased with *Miscanthus* growth in all treatment plots. Both mowing and fire significantly decreased the soil available N concentration in the 20–40 cm layer, but not in the 0–20 cm layer.

### 3.3 Bacterial and fungal community compositions with various treatments

A total of 882,695 bacterial and 1,159,799 fungal sequences were obtained from soils subjected to four treatments by high-throughput sequencing (**Table S1**). The sequences were classified into 1846 bacterial and 1663 fungal OTUs at a 97% sequence similarity cutoff (**Figure S1**). The 1846 bacterial OTUs obtained were divided into 10 phyla, and the 1663 fungal OTUs obtained were divided into 10 phyla. These bacterial and fungal rarefaction curves suggested that the 16S rRNA and ITS gene sequences for all samples reached the sequencing depths (**Figures S2A, B**).

**Figure 3:**
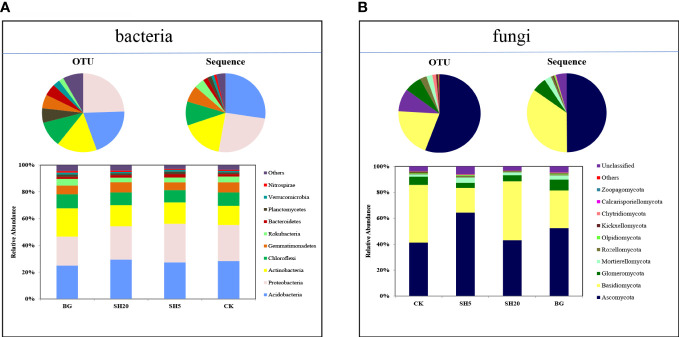
Taxonomic compositions (i.e., operational taxonomic unit [OTU], sequence, and relative abundance at the phylum level) of microbial community in soil samples under the CK, BG, SH5, and SH20 treatments: **(A)** bacteria and **(B)** fungi.

The relative abundances of bacterial and fungal communities at the phylum level were diverse among the CK, SH5, SH20, and BG treatments (**Figure 3**; **Table S1**). These four bacterial communities were dominated by Acidobacteria (372 OTUs, 27% sequences) (**Figure 1A**; **Table S1**), followed by Proteobacteria (452 OTUs, 25% sequences), Actinobacteria (292 OTUs, 17% sequences), and Chloroflexi (193 OTUs, 10% sequences) (**Figure 1A**; **Table S1**). In addition, the four fungal communities were dominated by *Ascomycota* (926 OTUs, 50% sequences) and *Basidiomycota* (337 OTUs, 35% sequences) (**Figure 1B**; **Table S1**). Relative to other treatments, SH5 showed a much greater increase in the relative abundance of Proteobacteria among all bacterial compositions but a decrease in the relative abundance of Basidiomycota among the fungal communities.

### 3.4 Bacterial and fungal community diversity of four treatments

**Figure 4:**
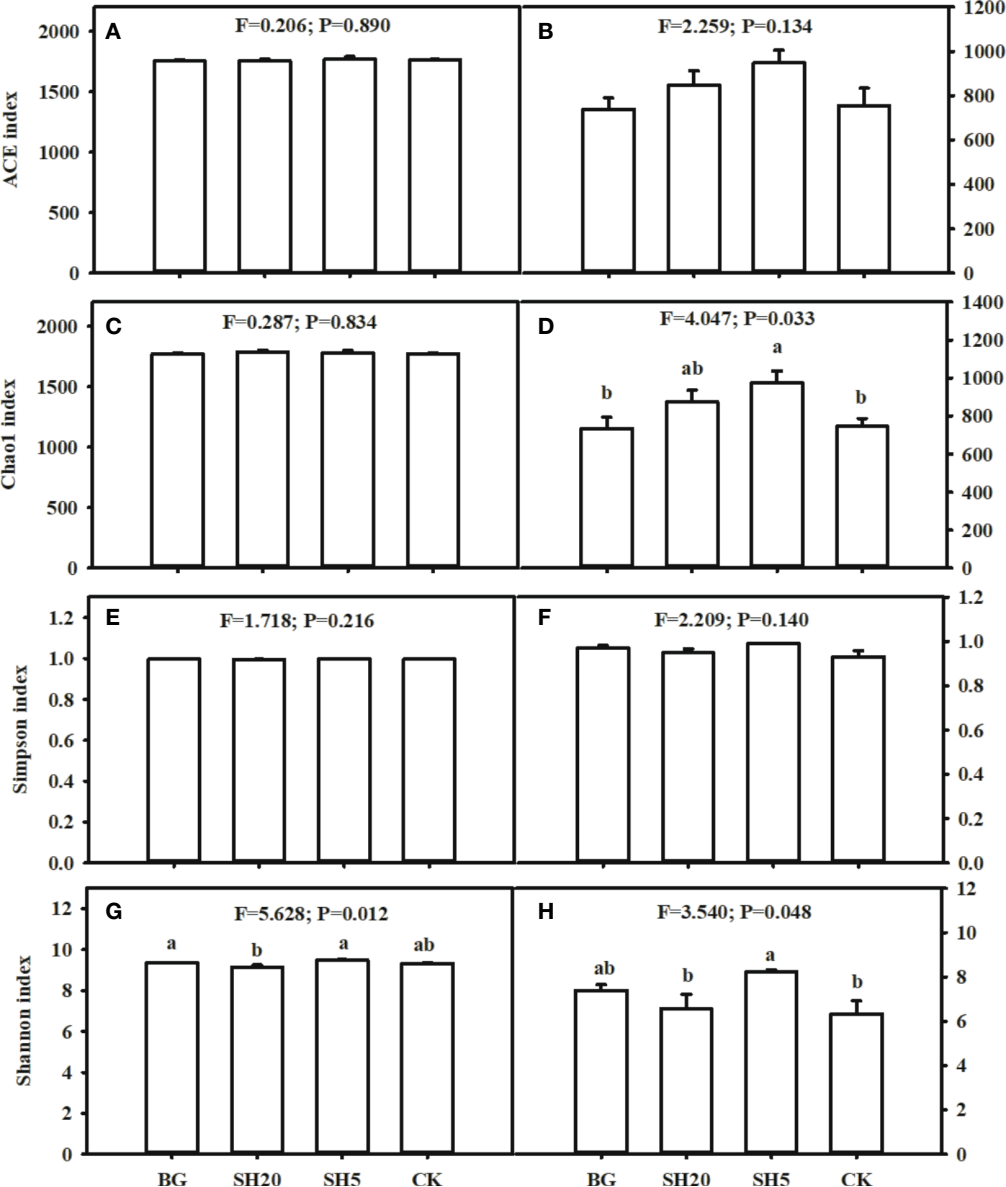
Alpha diversity index in the BG, SH20, SH5 and CK treatments. Left: Bacterial community diversity **(A, C, E, G)**; Right: fungal community diversity **(B, D, F, H)**. Within each subgraph according to Tukey’s mean separation test, values with the same lower case presents a non-significant difference (p = 0.05), while different lower-case letters show a significant (p < 0.05) difference.

The Simpson diversity, ACE, and Chao 1 richness indices of the four treatments showed no significant (p > 0.05) differences (**Figures 4A, C, E**), whereas the Shannon index was lower in SH20 than in the other three treatments, and this index among BG, SH5, and CK showed no significant (p > 0.05) difference (**Figure 4G**).

The ACE richness and Simpson diversity indices of the four fungal treatments showed no significant (p > 0.05) differences (**Figures 4B, F**). The Chao 1 indices of SH20 and SH5 were higher than those of CK and BG treatments, while there was no significant difference between SH20 and SH5 or between CK and BG (**Figure 4D**). The Shannon index of BG and SH5 was higher than that of SH20 and CK, while there was no significant difference between BG and SH5 or between SH20 and CK (**Figure 4H**).

**Figure 5:**
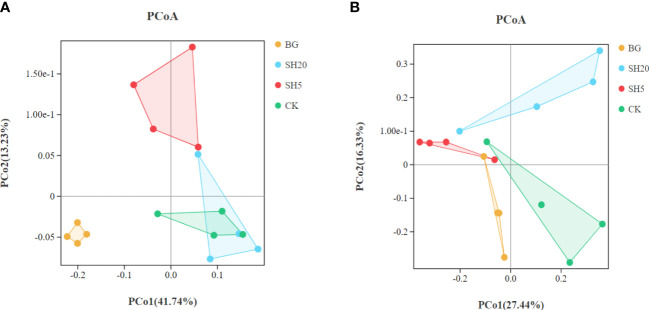
Principal coordinates analysis (PCoA) of the bacterial **(A)** and fungal communities **(B)** at the operational taxonomic unit (OTU) level based on Bray-Curtis dissimilarities.

Principal coordinate analysis (PCoA) showed that the bacterial community composition between BG and other treatments significantly (p < 0.05) differed (**Figure 5A**, **Table 2**). In addition, the fungal community composition between SH20 and the other treatments was significantly (p < 0.05) different (**Figure 5B**, **Table 2**).

**Table 2:** The statistical test of similarity (ANOSIM) and permutational multivariate two-way analysis of variance (PERMANOVA) to analysis the differences of bacterial and fungal community composition by amplicon sequencing.

Treatment		DF (degrees of freedom)	PERMANOVA		ANOSIM	
			Bray-Curtis		Bray-Curtis	
			*F*	*P*	*R*	*P*
Bacteria	T	3	2.849	0.0001	0.5174	0.0001
Fungi	T	3	3.215	0.0025	0.2821	0.0022

## 4 Discussion

Our experimental results positively highlight that both mowing and fire significantly (p < 0.01) reduced *Miscanthus* growth and dry material weight. This finding is consistent with results of previous studies on induced fire (Toma et al., 2010) and mowing (Clifton-Brown et al., 2017; Magenau et al., 2021). However, this effect was larger in the deeply mowed plots than in the burned plots. In particular, deep mowing induced a 5.2-fold decrease in dry plant biomass relative to the control, which is attributed to the significant alteration of plant growth traits, soil nutrient availability, and the community and structure of soil microorganisms.

**Figure 6:**
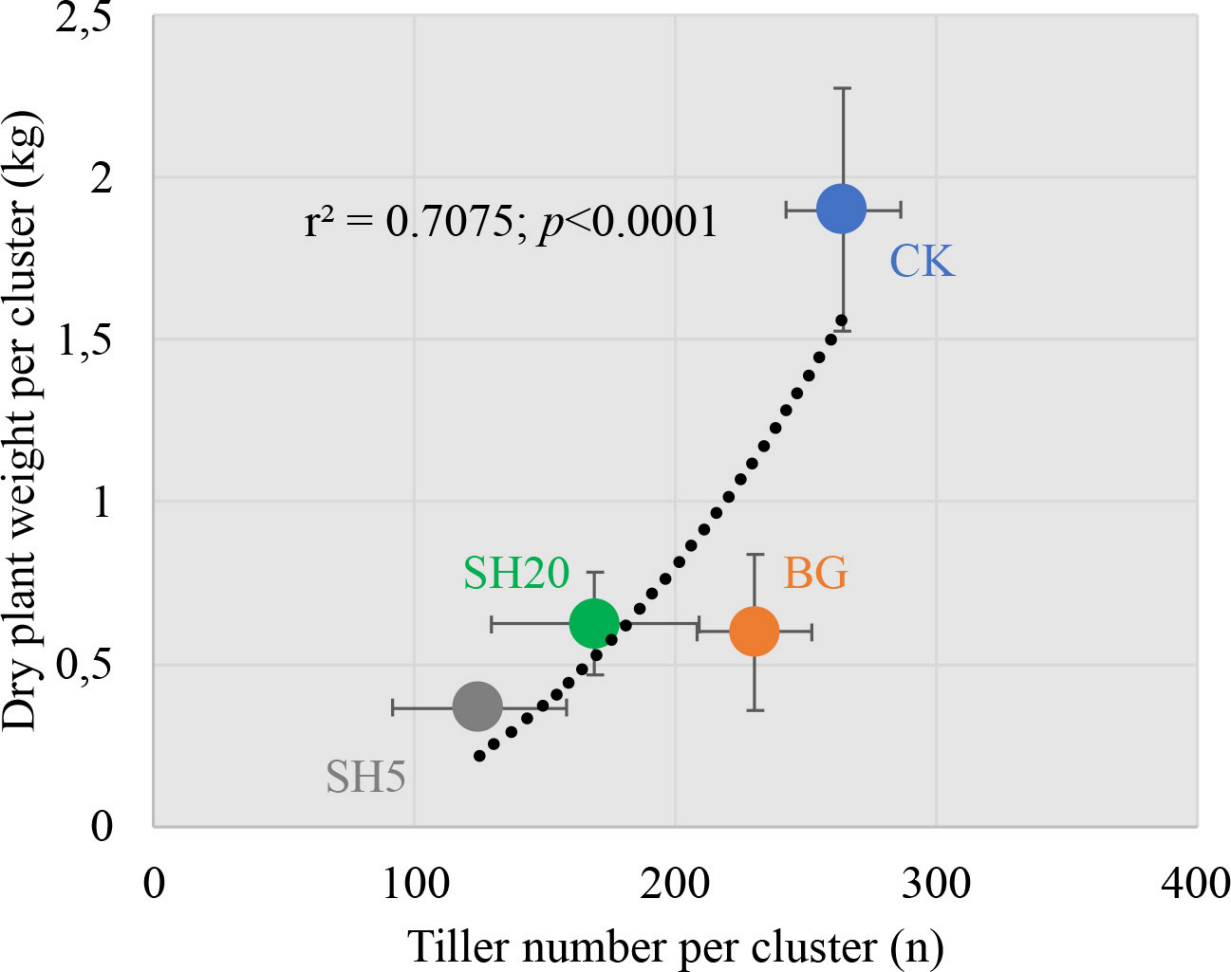
Plot of dry plant weight per cluster (kg/cluster) in four treatments against the tiller number per cluster (n).

First, after harvest, the tiller number per cluster in SH5 was the lowest among all treatments, as it showed a 2.2-fold decrease relative to that of the control (125 *vs*. 265); however, fire only induced a 1.1-fold decrease in the tiller number per cluster relative to that of the control (231 *vs*. 265). The decrease in tiller number in *Miscanthus* growth generally induced a decrease in aboveground dry weight, which is indicated by the significantly positive relationship between the dry plant weight per cluster and the tiller number per cluster (**Figure 6**). This finding is consistent with those of previous studies (Lee et al., 2017; Malinská et al., 2020). In addition, plant height in the deep-mowed plot was the lowest among all treatment plots. This could be directly affected by soil nutrient availability (Cadoux et al., 2012; Ivanyshyn et al., 2018) and will be discussed later.

Second, during *Miscanthus* growth, the soil nutrient availability (K and P) was much lower in both the mowed and burned plots than in the control, and this effect was much larger in the deep-mowed plot. This can be attributed to the removal of the aboveground straw by mowing, which directly leads to the loss of nutrients over their decomposition into soils (Kahle et al., 2001), similar to grasslands (Bakker et al., 2002; Hejcman et al., 2010; Magenau et al., 2021) and croplands (Buyanovsky and Wagner, 1986; DuPont et al., 2010; Cade-Menun et al., 2013). Fire also induces nutrient loss, as the burned soil is easily washed away by rain (Andreu et al., 1996; Thomas et al., 1999; Gimeno-García et al., 2000). As documented in many studies (Cadoux et al., 2012), soil nutrient availability is generally considered a key factor affecting *Miscanthus* growth (Himken et al., 1997). For example, in an alkaline grassland soil, both P and K deficiencies generally induced a decrease in *Miscanthus* (Roncucci et al., 2015; He et al., 2020). In particular, both mowing and fire during *Miscanthus* growth significantly induced a decrease in soil N availability in the 20–40 cm layer (**Figure 2**), which is the area of plant root activity for N uptake. Indeed, the role of N limitation as a factor determining successional processes in grass biomass production is significant, as the most striking result of previous studies to date is the significant response of plant growth to N fertilization (Van der Woude et al., 1994; Craine and Jackson, 2010; Toma et al., 2010; Whitaker et al., 2015; Zhao et al., 2016; Lee et al., 2017). If sustained, our current experimental results indicate that nutrient limitation (N, P, and K) is an important factor determining the rates of *Miscanthus* accumulation in repeated fires and mowed grasslands where there is a loss of soil nutrients.

**Figure 7:**
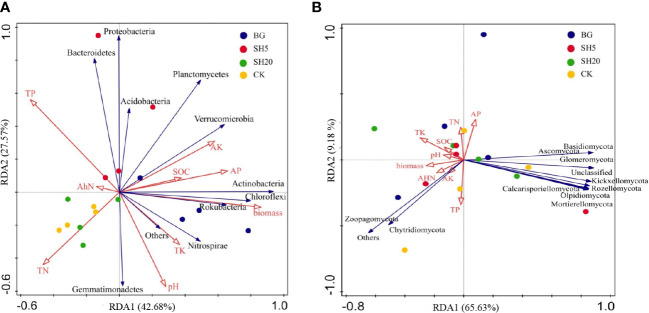
Redundancy analysis (RDA) of the correlations between soil microbial phylum (**A**: bacteria; **B**: fungi) and soil-plant variables under four treatments (CK, BG, SH5, and SH20). Soil-plant variables include soils factors (soil pH, total P [TP], total N [TN], total K [TK], organic carbon [SOC], available P [AP], available K [AK], and alkali-hydrolyzed N [AhN]) and a plant factor (dry biomass).

Our third hypothesis is that variations in bacterial and fungal composition are altered by fire and mowing, which indirectly alters other soil factors, such as nutrient and micronutrient availability. Soil microbial communities play a crucial role in regulating soil nutrient availability (Cheplick et al., 1989; McCaig et al., 1999; Unger et al., 2013), suggesting that their interaction determines soil sustainability and the development and productivity of plants (Song et al., 2021). Mowing and fires alter the source of energy and nutrients for soil microbial growth, as illustrated in this study (**Table 1**; **Figures 1**–**4**), in which both fire and mowing redistributed the community composition and diversity of soil bacteria and fungi. This redistribution is much greater in the deep mowing than in the BG, which could be a crucial factor resulting in the diffidence of *Miscanthus* aboveground biomass. The coverage, richness, and diversity of the soil microbial community were well isolated in soils with four grassland managements, according to three alpha diversity indices (**Figure 3**; **Figure S1**). Thus, our experimental findings indicate that deep mowing slightly altered the composition of soil bacteria, but largely altered the composition of soil fungal communities relative to other treatments (**Figure 3**). This finding is consistent with previous research showing the responses of soil bacterial and fungal communities to various grassland mowing and management practices (Cui et al., 2020). This effect can be attributed to two factors. Soil microbial communities are sensitive to variability in mowing dryland ecosystems on a global scale owing to changes in nutrient sources (Delgado-Baquerizo et al., 2017). Deep mowing exacerbates the negative impacts of soil water loss on soil microbial communities (Blagodatsky and Smith, 2012). Moreover, this practice exacerbates the loss of soil nutrients (**Figure 2**) due to the greater loss of the litter biomass (i.e., a much lower stubble heights-induced by the deep mowing) relative to other treatments. Therefore, the bacteria and fungi were significantly altered by deep mowing compared to other treatments because of soil environmental conditions and nutrients. First, deep mowing over *Miscanthus* growth significantly decreased soil OC and N (**Table 1**), which can be used to provide a direct energy source and nutrients to soil microbes, affecting their growth and abundance. This study also observed that in SH5, soil microbial abundance (e.g., Firmicutes, and especially Proteobacteria) increased relative to that of the control (**Figure 3**). This is consistent with previous results, demonstrating that *Proteobacteria* was abundant in less healthy soils (Li et al., 2014), as its abundance is sensitive to soil nutrient status (Esperschütz et al., 2007; Unger et al., 2013). This induced alteration in deep mowing significantly led to a decrease in the abundance of *Basidiomycota* and an increase in Ascomycota among the fungal communities. In particular, it further illustrated a much greater effect on fungal community diversity at the level of alpha diversity index (considering the Simpson diversity, ACE, and Chao 1 richness indexes) than other treatments (**Figures 4**, **5**). Second, due to the loss of key energy and nutrients induced by plant growth, the soil microbial community diversity was altered. This study highlights that the sources of N, K, and P in the deep mowing over *Miscanthus* growth significantly decreased (**Table 1**); thus, the soil microbial community diversity was significantly altered (**Figures 3**, **4**). However, the reason for this remains unclear and needs to be further investigated to determine the differences between bacteria and fungi in soils with poor fertility. More interestingly, the factors of soil pH, available P and K, total N (TN), alkali-hydrolyzed N (AhN), and total OC explained 43% of soil bacterial community and 66% of soil fungal community (**Figure 7**), highlighting their importance for soil microbial communities. In particular, for SH5, Proteobacteria and Acidobacteria at the phylum level were significantly related to the sources of energy and nutrients, including SOM, total N and P, and available N and P (**Figure 7A**). Similarly, the soil factors in SH5 were significantly related to those in most fungal communities. As discussed above, our experimental results clearly indicated that deep mowing resulted in a higher redistribution of the soil microbial community among all treatments. Although the stability of microbial communities in grasslands significantly affects soil quality and immunity (McCaig et al., 1999; Bonkowski and Roy, 2005; Otto et al., 2005; Cui et al., 2020), the structure and function of microbial communities in dry grasslands are influenced by numerous physicochemical soil factors (Jangid et al., 2011; Griffiths and Philippot, 2013; Zheng et al., 2019). Microbial communities are, therefore, regarded as a crucial mechanism affecting soil quality, which supports our third hypothesis. The use of various grassland managements has already been reported to alter the species abundance and composition of the soil microbial community during grass growth by altering soil nutrient availability, thereby affecting the aboveground biomass, as illustrated in **Table S2**, which shows that biomass best explained the interaction between soil quality and microbial community. Here, our results demonstrate that the composition of the soil microbial community is significantly affected by deep mowing by altering soil chemical properties (especially soil N and P availability, as well as SOC), indirectly driving *Miscanthus* productivity.

## 5 Conclusion

Both fire and mowing significantly decreased the soil available N, P, and K content, tiller number per cluster, and plant height during *Miscanthus* growth relative to the control. However, these effects were greater in the deep-mowing plot than in the other plots. After harvest, both fire and mowing significantly decreased the dry weight of *Miscanthus*, whereas deep mowing induced a 5.2-fold decrease in dry plant biomass relative to the control. This could be attributed to the removal of aboveground straw by mowing, which directly leads to the loss of nutrients in soils. If sustained, our current experimental results indicate that nutrient limitation (N, P, and K) was an important factor determining the rates of *Miscanthus* accumulation in the repeated fires and the deep-mowing grasslands due to the loss of soil nutrients. In addition, both fire and mowing redistributed the community composition and diversity of the soil bacteria and fungi. This redistribution was much greater in deep mowing than in fire and light mowing. Soil bacterial abundance (e.g., Firmicutes, and especially Proteobacteria) in the deep-mowing plot increased relative to that of the control; this is consistence with previous results, indicating that Proteobacteria are abundant in less healthy soils as they are sensitive to the soil nutrient status. In contrast, deep mowing led to a significant decrease in the abundance of Basidiomycota and an increase in Ascomycota among the fungal communities, whereas it had an even greater effect on fungal community diversity at the level of alpha diversity indexes (considering the Simpson diversity, ACE, and Chao 1 richness indices) than other treatments. Thus, the variation in bacterial and fungal composition was altered by deep mowing, which indirectly altered other soil factors such as nutrient and micronutrient availability, resulting in a decrease in plant productivity. This finding indicates that deep mowing, rather than fire, induces soil processes and health development, thus restraining grass sustainability in a dry-alkaline grassland.

## Data availability statement

The datasets presented in this study can be found in online repositories. The names of the repository/repositories and accession number(s) can be found in the article/**Supplementary Material**.

## Author contributions

ZM: Experiment; data collection, writing the original draft, and analysis. YW: Editing, formal analysis, and made suggestions of the manuscript. QL and WL: Experiment and editing. HW and YL: Experiment, editing, and analysis. MY: Conceptualization, editing, made suggestions of the manuscript, and corresponding author. All authors contributed to the article and approved the submitted version.
