# Supplementary material for: Deep mowing rather than fire restrains grassland Miscanthus growth via affecting soil nutrient loss and microbial community redistribution
Source: Front Plant Sci. 2023 Jan 13;13:1105718. doi: 10.3389/fpls.2022.1105718 (PMC9880543; doi:10.3389/fpls.2022.1105718)
Supplement: Supplementary file 1 [file DataSheet_1.docx]

1. (B)


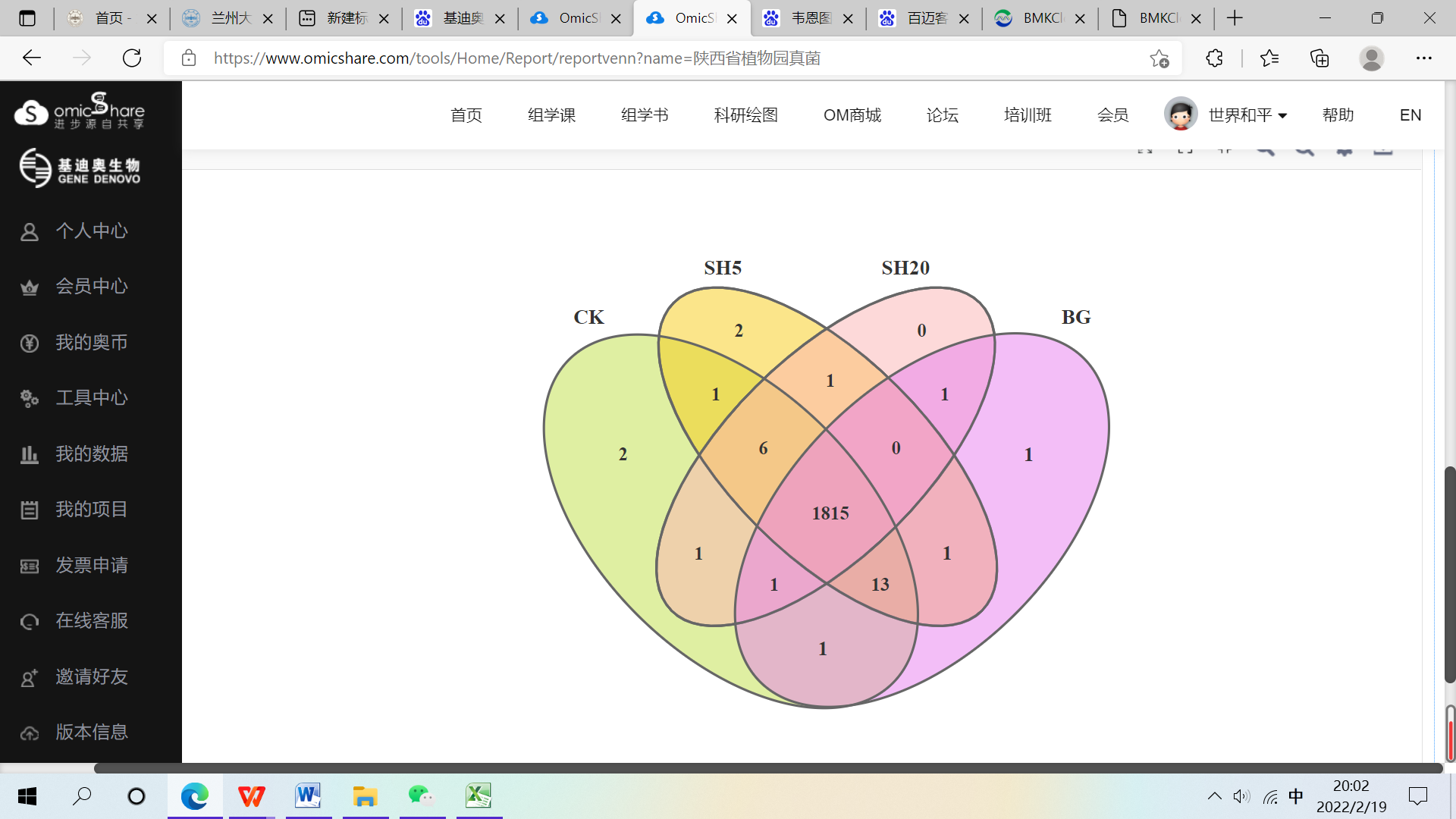

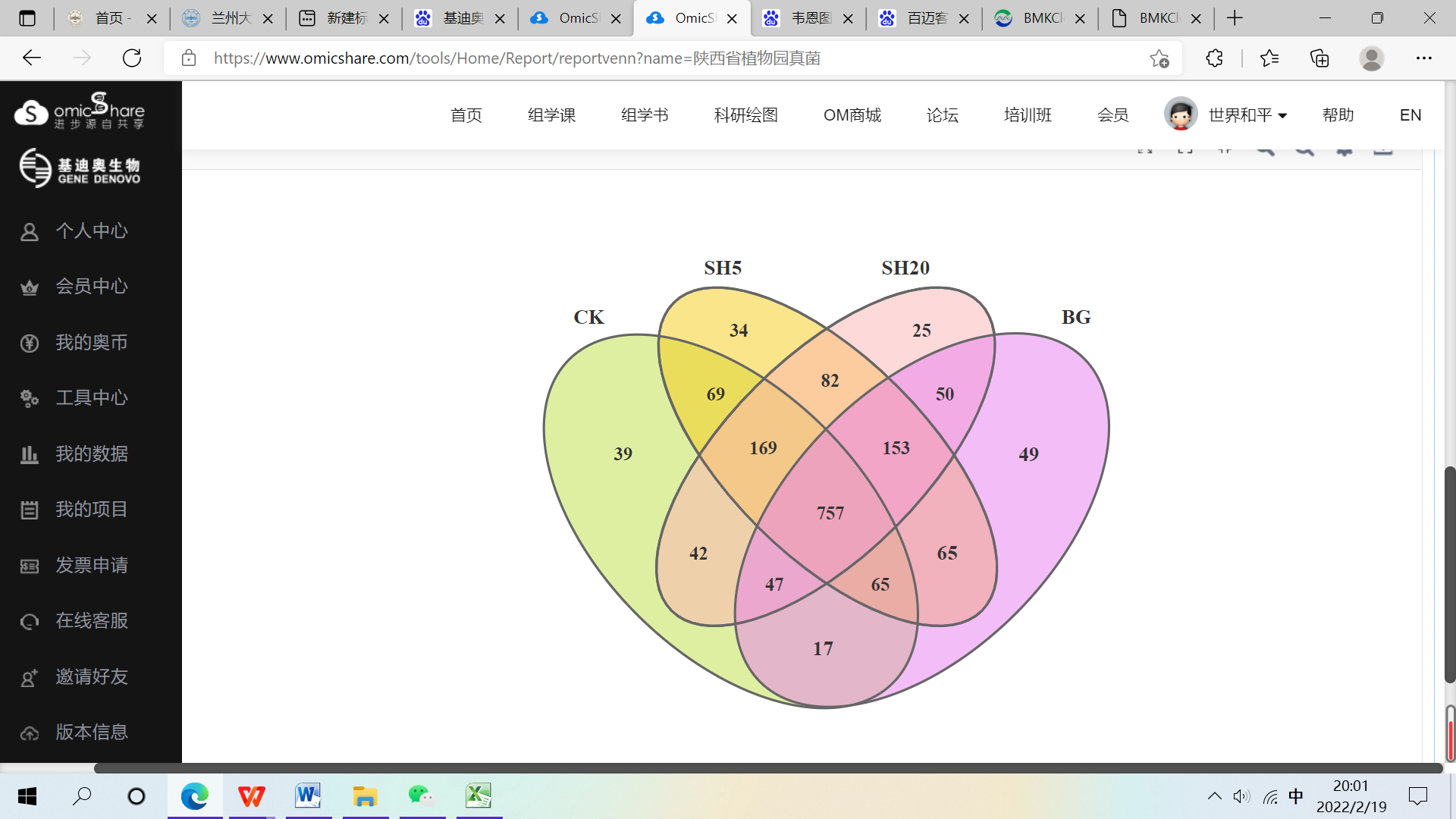


**Figure S1**. Venn diagram of bacterial (A) and fungal (B) OTUs under the treatment of CH, SH5, SH20 and BG.

1. (B)


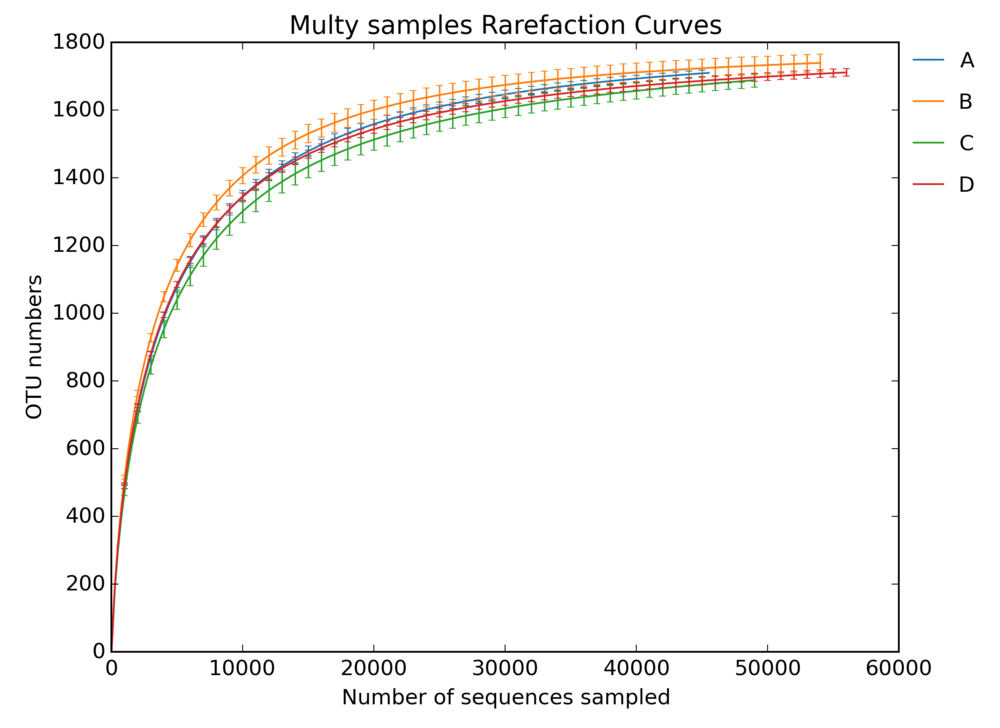

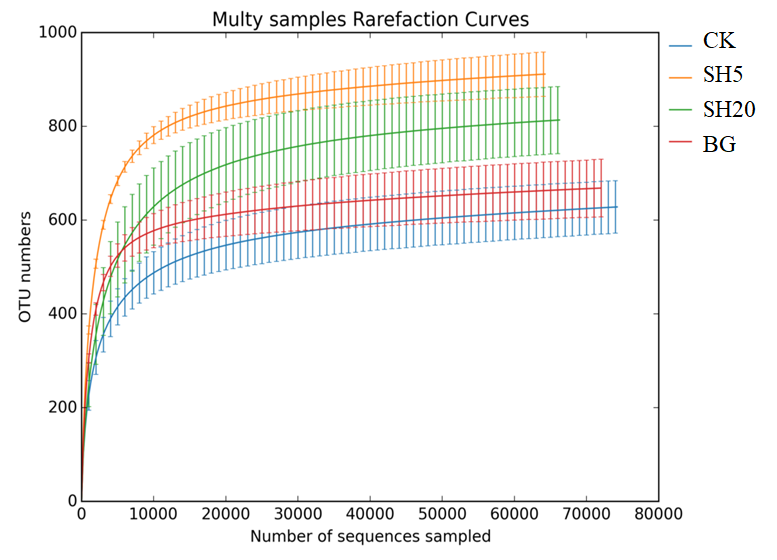


**Figure S2.** Rarefaction curves of the 16S rRNA and ITS gene sequence of bacteria (A) and fungi (B) under the treatment of CK, SH5, SH20 and BG.

**Table S1.** Bacterial and fungal community composition of CH, SH5, SH20 and BG treatments at the phylum level.

| Relative abundance (%) | | | | | | | |
| --- | --- | --- | --- | --- | --- | --- | --- |
| Microbes | Phylum | CK | SH5 | SH20 | BG | OTU | Sequence |
| Bacteria | Acidobacteria | 0.28 | 0.27 | 0.29 | 0.25 | 372 | 241470 |
|  | Proteobacteria | 0.27 | 0.29 | 0.25 | 0.22 | 452 | 224959 |
|  | Actinobacteria | 0.14 | 0.16 | 0.16 | 0.21 | 292 | 149995 |
|  | Chloroflexi | 0.10 | 0.09 | 0.09 | 0.11 | 193 | 86370 |
|  | Gemmatimonadetes | 0.08 | 0.06 | 0.08 | 0.07 | 104 | 60338 |
|  | Rokubacteria | 0.04 | 0.04 | 0.03 | 0.05 | 35 | 36156 |
|  | Bacteroidetes | 0.02 | 0.02 | 0.02 | 0.02 | 89 | 17076 |
|  | Planctomycetes | 0.01 | 0.02 | 0.01 | 0.02 | 110 | 14575 |
|  | Verrucomicrobia | 0.01 | 0.01 | 0.01 | 0.01 | 51 | 10313 |
|  | Nitrospirae | 0.01 | 0.01 | 0.01 | 0.01 | 11 | 8137 |
|  | Others | 0.03 | 0.03 | 0.04 | 0.04 | 137 | 33306 |
|  | Total | 100 | 100 | 100 | 100 | 1846 | 882695 |
| Fungi | Ascomycota | 0.41 | 0.64 | 0.43 | 0.52 | 926 | 579093 |
|  | Basidiomycota | 0.44 | 0.19 | 0.45 | 0.29 | 337 | 404235 |
|  | Glomeromycota | 0.06 | 0.04 | 0.05 | 0.08 | 114 | 68382 |
|  | Mortierellomycota | 0.02 | 0.04 | 0.02 | 0.03 | 39 | 32188 |
|  | Rozellomycota | 0.01 | 0.01 | 0.01 | 0.01 | 44 | 9623 |
|  | Olpidiomycota | 0.01 | 0.01 | 0.00 | 0.01 | 3 | 5505 |
|  | Kickxellomycota | 0.01 | 0.00 | 0.00 | 0.00 | 14 | 5593 |
|  | Chytridiomycota | 0.00 | 0.01 | 0.00 | 0.00 | 25 | 3566 |
|  | Calcarisporiellomycota | 0.00 | 0.00 | 0.00 | 0.00 | 1 | 552 |
|  | Zoopagomycota | 0.00 | 0.00 | 0.00 | 0.00 | 1 | 115 |
|  | Others | 0.00 | 0.00 | 0.00 | 0.00 | 3 | 246 |
|  | Unclassified | 0.04 | 0.06 | 0.03 | 0.05 | 156 | 50701 |
|  | Total | 100 | 100 | 100 | 100 | 1663 | 1159799 |

**Table S2**. Analytical results of ‘Interactive-forward-selection’. Note: Soil-plant variables includes soils factors (soil pH, total P (TP), total N (TN), total K (TK), organic carbon (SOC), available P (AP), available K (AK), and alkali-hydrolyzed N (AhN)) and plant factor (dry biomass).

| Microbes | Name | Explains % | Contribution % | pseudo-F | P |
| --- | --- | --- | --- | --- | --- |
| **Bacteria** | Biomass | 32.2 | 39.6 | 6.7 | 0.002 |
|  | pH | 14.5 | 17.8 | 3.5 | 0.006 |
|  | TP | 7.6 | 9.3 | 2.0 | 0.1 |
|  | TN | 4.7 | 5.7 | 1.3 | 0.308 |
|  | AK | 3.0 | 3.6 | 0.8 | 0.564 |
|  | SOC | 2.3 | 2.8 | 0.6 | 0.642 |
|  | TK | 6.4 | 7.8 | 1.7 | 0.156 |
|  | AP | 8.2 | 10.0 | 2.7 | 0.078 |
|  | AHN | 2.6 | 3.2 | 0.8 | 0.452 |
| **Fungi** | TK | 6.1 | 7.7 | 0.9 | 0.352 |
|  | AHN | 14.7 | 18.4 | 2.4 | 0.136 |
|  | AP | 6.4 | 8.0 | 1.1 | 0.316 |
|  | TN | 9.3 | 11.6 | 1.6 | 0.208 |
|  | pH | 5.7 | 7.2 | 1.0 | 0.358 |
|  | TP | 20.8 | 26.0 | 5.1 | 0.038 |
|  | SOC | 11.6 | 14.6 | 3.7 | 0.06 |
|  | AK | 3.1 | 3.9 | 1.0 | 0.412 |
|  | biomass | 2.1 | 2.6 | 0.6 | 0.528 |
